# Case Report: Lesion network guided transcranial direct current stimulation targeting treatment refractory hallucinations and delusions: a traditional and accelerated stimulation case study

**DOI:** 10.3389/fpsyt.2025.1568895

**Published:** 2025-05-08

**Authors:** Nicolas Raymond, Rebekah Trotti, Paulo Lizano

**Affiliations:** ^1^ Department of Psychiatry, Beth Israel Deaconess Medical Center, Boston, MA, United States; ^2^ Division of Translational Neuroscience, Beth Israel Deaconess Medical Center, Boston, MA, United States; ^3^ Department of Psychiatry, Harvard Medical School, Boston, MA, United States

**Keywords:** lesion network mapping, superior temporal sulcus, auditory hallucinations, treatment resistance, EEG, transcranial electric current stimulation

## Abstract

Transcranial electrical stimulation (tES) has been shown to reduce symptoms related to psychosis, especially positive symptoms such as auditory hallucinations (AH). However, there are time and transportation burdens that fall on patients since typical tES treatments are performed over 5 days in-clinic and consist of twice daily tES sessions. Evidence suggests that accelerated protocols (repeated number of tES sessions over fewer days) may have similar efficacy as traditional 5-day tES protocols. Moreover, few investigations have sought to target novel brain regions linked to AH, such as those identified by advanced neuroimaging studies that identify causal neural substrates that manifest AH. Here, we report on a 62-year-old woman with persistent treatment-resistant AH. We performed two tES treatment protocols (a 5-day traditional protocol and a 2-day accelerated protocol) targeting a brain region that has been causally linked to the manifestation of AH, the right superior temporal sulcus (rSTS). Both traditional and accelerated protocols resulted in a decrease in AH and distinct electroencephalogram (EEG) changes that tracked with symptom changes.

## Introduction

Auditory hallucinations (AH) are a core feature of psychosis spectrum disorders (PSD) and other neuropsychiatric disorders (1). While antipsychotics are considered first line treatment for these symptoms, upwards of 20 percent of individuals fail to respond adequately to these medication interventions (2). Moreover, antipsychotics can produce significant side effects including metabolic dysregulation, tardive dyskinesia and sedation (3).

On the other hand, noninvasive brain stimulation (NIBS) interventions such as transcranial magnetic stimulation (TMS) have shown some success in controlling AH (4) and other symptoms of PSD (5). In comparison to antipsychotics, TMS and other forms of NIBS have low side effect profiles (6, 7). However, TMS has a significant overhead cost compared to other forms of NIBS (8). tES technologies, on the other hand, are relatively inexpensive, mobile, and may have a similar efficacy profile when compared to TMS (9). tES has been shown to reduce symptoms related to PSD such as negative symptoms (10) and positive symptoms including visual hallucinations (11). One form of tES, transcranial direct current stimulation (tDCS), may be efficacious for the management and treatment of AH; however, conflicting results exist in the literature (12). This may be due to lack of sufficient tES dosing parameters (13) and lack of an optimal neural target to causally reduce AH manifestation.

Typically, many days (5 days, twice daily) of tES are needed to achieve an effect, posing a significant burden to patients. However, some forms of NIBS like TMS have shown that accelerated protocols are efficacious and may possibly contribute to compounding effects of stimulation. Accelerated protocols collapse the standard number of sessions into fewer days, which could maximize neuroplastic effects. Furthermore, shorter intervals between sessions may be more effective than long intervals (3-24 hours) (14) by maximizing the effect of tES on long term potentiation. In addition, accelerated protocols would drastically decrease the burden of patients traveling to lab settings over the traditional 5 day treatment period. Moreover, a recent dose-response meta-analysis suggested even more sessions are needed (48-72 coulombs for an effective dose of 50%- 95%), relative to the common 24 coulomb dose (15) to achieve a desired clinical outcome. To achieve these dosing requirements, accelerated protocols may be particularly helpful.

While the literature at hand has primarily focused on the left temporal parietal junction or fronto-networks as primary targets for tES in relation to AH, a recent causal lesion network mapping study identified the rSTS target, which has negative connections to 90% of brain lesions causing hallucinations, regardless of sensory modality (16). Utilizing regions of interests (ROIs) to stimulate from casual lesion network mapping studies has shown some success in past studies (17). Moreover, the rSTS has a distinct role in sensory integration (18), is hyperactive in PSD, and is associated with psychosis proneness (19).

EEG, also termed “quantitative EEG” or qEEG, is being increasingly utilized in tracking the progression of psychiatric disease and can be easily incorporated into tES protocols. Specifically, quantitative measures obtained from resting state EEG differ from healthy controls in a broad range of disorders with substantial within-disorder variability (20). In psychosis research, EEG has been utilized in tracking medication response (21), detecting individuals at high-risk (22) and predicting response to noninvasive brain stimulation (23). While there is still no definitive resting state EEG biomarker that determines psychiatric diagnosis or symptomatic state, more comprehensive investigations and stringent replicability standards in the field will benefit from studies employing EEG to characterize disease state and tES treatment response. There is a growing consensus that EEG may have a more viable future in clinical psychiatry practice given its low-cost profile, scalability and at-home capabilities (24). Moreover, it has been shown that tES and EEG can feasibly be combined for at-home treatment (25).

Thus, there is a need to develop novel accelerated protocols with the appropriate dosage and further establish the safety, feasibility, efficacy, and burden profile for patients with PSD and their caregivers. Here, we present the first accelerated high definition-tDCS (HD-tDCS) protocol for targeting hallucinations in PSD. The subject in this case study participated in an open label arm of a larger sham-controlled trial testing HD-tDCS efficacy (2x20min for 5 days) delivered to the right superior temporal sulcus (rSTS) to reduce hallucinations. We primarily present this case to document protocol feasibility and test extension to an accelerated protocol, with the intent to more rigorously test the clinical efficacy of rSTS stimulation in the ongoing placebo-controlled trial. The study was approved by the Institutional Review Board at Beth Israel Deaconess Medical Center and the participant provided their informed consent.

## Case description

This is a case of a 62-year-old Spanish-speaking female with a 30-year history of postpartum onset schizophrenia, multiple comorbid cardiometabolic diseases, and a right periventricular corona radiata/subcortical parietal infarct. She experienced treatment refractory symptoms: multimodal hallucinations (primarily auditory and visual), delusions (primarily paranoid, religious, and grandiose), high excitement, conceptual disorganization, hostility, emotional withdrawal, stereotyped thinking, anxiety, uncooperativeness, and impaired insight. Her caregiver reported the hallucinations, delusions, and self-dialogue were most impairing since it was difficult to take her on errands, maintain clinical visits and medication compliance, and get appropriate sleep.

Symptoms persisted for many years despite stable doses of lurasidone (160mg daily), olanzapine (20mg nightly), and divalproex DR (250mg daily), which produced significant side effects including sedation and cardiometabolic derangements. Past unsuccessful medication trials involved multiple other antipsychotics including clozapine and other medication combinations. All failed to adequately control their symptoms or improve the quality of life for the participant or their loved ones. Prior to enrollment in our protocol, the participant was on stable doses of lurasidone (120mg daily), sertraline (200mg daily), and lamotrigine (400mg daily).

## Study description and findings

*Diagnostic Assessment.* To partake in the trial, informed consent was provided in Spanish, the participant’s preferred language. A full medical history and psychiatric assessments including the 7-Item Auditory Hallucination Rating Scale (AHRS) (26) and Positive and Negative Syndrome Scale (PANSS) (27) were collected. The patient’s caregiver (adult daughter) provided collateral information on symptoms and medical history. Data was also collected via chart review.

**Figure 1:**
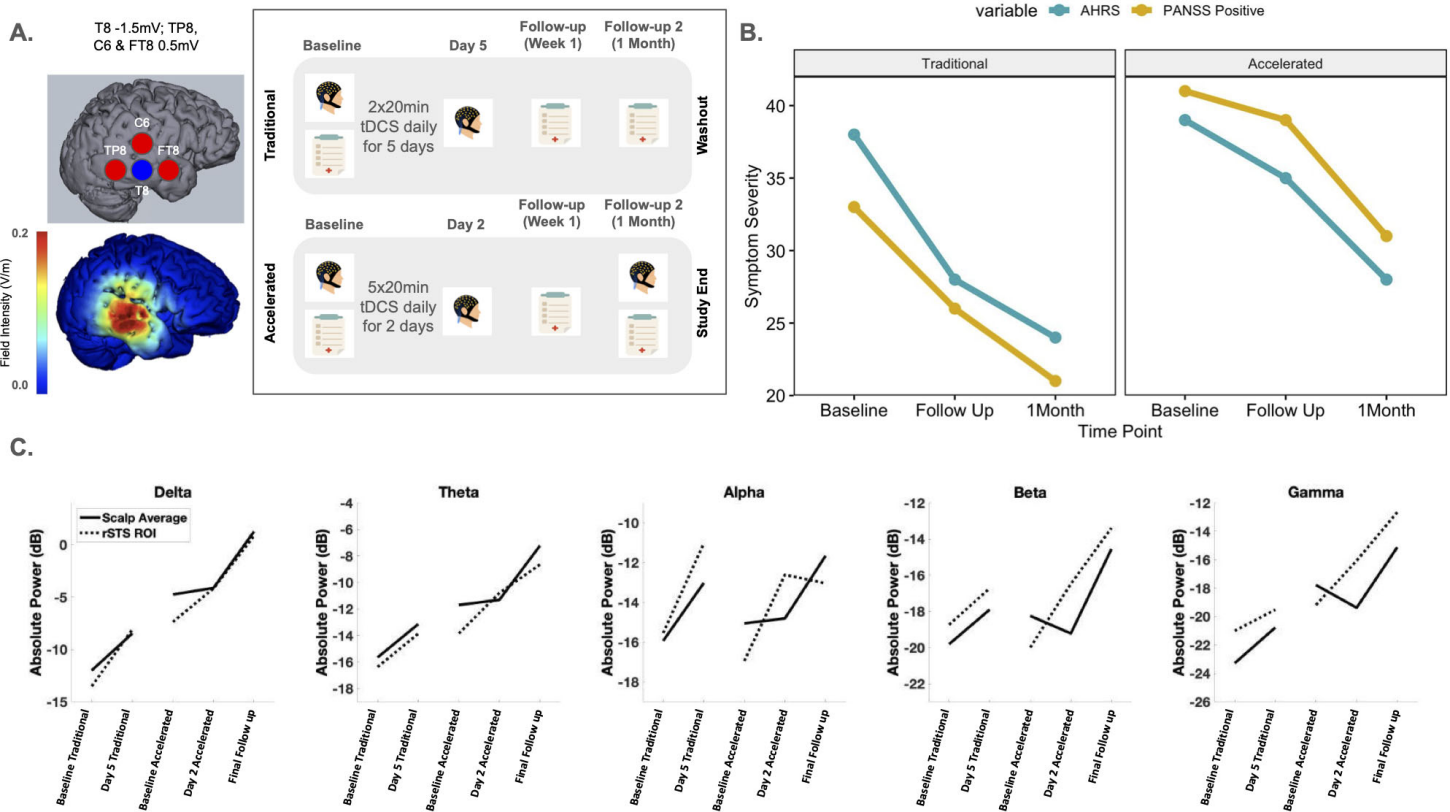
**(A)** Sensor layout and current intensity at each sensor location for our tDCS montage targeting the right superior temporal cortex and current flow model. The study timeline for the study assessments (EEG and clinical assessments) and the daily tDCS treatment received for both the Traditional and Accelerated protocols are shown. **(B)** Raw scores calculated for AHRS and PANSS Positive variables over time (Baseline, Follow up and 1 Month assessments) for both Traditional and Accelerated protocols. **(C)** Absolute power values for each frequency band over the time course of the study. tDCS, transcranial direct current stimulation; AHRS, Auditory Hallucination Rating Scale; PANSS, Positive and Negative Symptom Scale; EEG, electroencephalogram.

*Therapeutic Intervention.* The 2 study arms employed 10 sessions of HD-tDCS to the rSTS as follows: 1) a traditional 5 day, 2x daily protocol and 2) an accelerated 2 day, 5x daily protocol. Each session lasted 20 minutes. **Figure 1A** contains full montage, current flow modeling, and timeline details. Protocols were delivered using the Soterix MXN-9 High Definition-Transcranial Electrical Current Stimulator, Model 9002 A. Between protocols, a >12-week washout period was completed.

*Outcomes.* The AHRS (26) and PANSS (27) were conducted with collateral information from the caregiver to determine symptom severity and clinical response at each timepoint (Baseline; Follow up 1; Follow up 2) (**Figure 1B**). AHRS Total scores were calculated from the scale’s seven domains (hallucination frequency, reality, loudness, number of voices, length of hallucinations, attentional salience, and distress level) which capture AH activity for the past 24 hours. PANSS Positive scores were calculated from the subscale within the PANSS which consists of 7-items scored between 1 (absent) to 7 (extreme).

To measure acute effects of stimulation on neurophysiology, 5 minutes of eyes-open resting state EEG were also collected before and after stimulation was completed for both protocols (day 5 for the traditional protocol and day 2 for the accelerated protocol). EEG was preprocessed using the Harvard Automated Preprocessing Pipeline for Electroencephalography (HAPPE; (28)) and transformed to frequency data using a fast Fourier transform (FFT). We examined topographical distributions and whole-scalp averages of EEG absolute power (in dB) at the delta (2-4 Hz), theta (4-8 Hz), alpha (8-12 Hz), beta (12-30 Hz), and gamma bands (30-100 Hz). In addition, we derived a rSTS region of interest (ROI) (See **Supplementary Figure S2**), to detect changes related to the rSTS specifically. The electrodes that were chosen for the rSTS ROI best reflected the electrodes that were chosen in the stimulation montage.

Clinical follow ups were performed 1 week and 1 month after stimulation began for both protocols. EEGs were collected at baseline visits and after tDCS treatment was finished to measure acute effects. Lastly, a final EEG was collected at the last visit during the 1 month accelerated protocol follow up.

*Timeline.* The first baseline clinical and EEG assessment took place in March 2024. After the first assessments were completed, the traditional protocol of 5 days, 2x daily (20min) of HD-tDCS was delivered. After a 3 month washout period, the accelerated protocol consisting of 2 days, 5x daily (20min) of HD-tDCS was implemented in August 2024. The final follow up clinical assessment including EEG took place in September 2024.

*Results.* PANSS and AHRS scores showed symptom reduction following the traditional and accelerated protocols (**Figure 1B**). Specifically, PANSS positive and general scores decreased with stimulation. AHRS showed reductions in hallucination frequency, loudness, attentional salience, and distress. During the washout periods, symptoms returned to baseline level. After the accelerated protocol, symptoms decreased in a similar manner as before. Traditional tDCS had a more pronounced therapeutic effect between baseline and follow up 1 compared to accelerated; however, the accelerated protocol had a more pronounced effect compared to traditional tDCS between follow up 1 and the 1 month time point (**Supplementary Figure S1**). The patient’s caregiver reported significant improvement in the patient’s and family’s quality of life. The patient’s sleep, hallucinations, delusions, and self-dialogue improved. She also became more redirectable and engaged with the family. However, insight and judgment did not improve.

EEG data showed increased power across all frequency bands (delta, theta, alpha, beta, and gamma; **Supplementary Figure S3**) after both protocols were completed compared to the first baseline EEG, though we expected stimulation to reduce power. During the washout period between the traditional and accelerated protocols, alpha and beta values returned to baseline like values for both the whole head and rSTS ROI. However, delta, theta and gamma power values continued to remain higher at the start of the accelerated protocol. In addition, these power values continued to increase until the final follow up. Interestingly, while both the rSTS ROI and whole head had an acute power increase in the traditional protocol, the acute effect only occurred at the rSTS in the accelerated protocol. The whole head only showed a power increase at the final follow up, suggesting a delayed response from the accelerated tES treatment (**Figure 1C**). It may be the case that tES shows its greatest effect locally and then these effects began to alter larger scale brain networks later in time. In relation to symptoms, it is important to note that AHRS and PANSS Positive scores saw a stark increase between protocols. This may be related to the decrease in alpha and beta values between protocols.

Overall, both protocols were well tolerated and sensations were measured by short questionnaires related to sensations. Motor instability was present prior to the study and slightly worsened in the first 3 minutes following stimulation. HD-tDCS could have exacerbated this due to the proximity of the rSTS to the motor cortex or may have been the result of dizziness, but resting and moving cautiously immediately following stimulation adequately addressed this concern. No contact dermatitis (29) or other adverse events/sensations were observed or reported after any stimulation protocol.

## Discussion

This case report highlights the potential utility of HD-tDCS to manage treatment resistant hallucinations/delusions while reducing significant side effects and supports the feasibility of study protocols that reduce participant burden, as accelerated protocols may address time constraints and accessibility concerns. The traditional and accelerated protocols showed equivalent safety and efficacy profiles in this case study.

In conjunction, the EEG results suggest that accelerated protocols may achieve a similar increase in oscillatory power in comparison to traditional tDCS protocols. Some literature has shown that greater activation of the default mode networks is present during hallucination free episodes (30). Our EEG results from this case show a similar increase in neural activity as symptoms reduced. However, medications have been shown to contribute to changes in EEG measures for those with psychosis (31). Thus, the EEG results should be interpreted with caution as the patient’s medication could have resulted in distinct EEG changes. However, given the increase of power values seen in both the traditional and accelerated protocol, the effect of tES may still be the contributing factor for this change.

Currently, few studies have employed an accelerated transcranial direct current stimulation (tDCS) protocol in PSD and in this case report was well tolerated and achieved a similar efficacy profile compared to traditional tDCS protocols (32, 33). Both these studies did not incorporate EEG as an adjunct measurement of brain activity. There are significant limitations in the current case report including the lack of sham protocol to corroborate results. In addition, while there was a washout period between tES protocols, there could be a compounded effect of the traditional tES treatment on the accelerated tES treatment; however, literature suggests that the effects of tDCS do not last long on symptoms (34). However, this is an area of research that needs further investigation to identify the mechanism of tES on cortical excitability. In addition, these effects are most likely dependent on varying factors including brain region targeted as well as stimulation parameters. However, we believe this case study is a significant contribution to the current literature given the novelty of the ROI selected, the incorporation of high-density EEG and comparison of traditional vs accelerated protocols. Moreover, given the past failed medication interventions in controlling the patient’s AH, this gives additional weight to the tDCS treatment employed in the protocol. Lastly, while not considered in this case report, individual EEG characteristics including baseline activity as well as disease state should be considered in future trials. It has been shown that tES response may be reliant on individual brain characteristics (35–37) and that brain characteristics of those with psychosis may inhibit the effects of tES resulting in potential increased dosing requirements to achieve a desired effects (38). Overall, our case study builds on this literature regarding the clinical efficacy of HD-tDCS in the management of treatment refractory cases and provides more evidence of a potential EEG biomarker to consider in future large-scale trials.

## Data Availability

The raw data supporting the conclusions of this article will be made available by the authors, without undue reservation.
