# Supplementary figures and images for: Case Report: Lesion network guided transcranial direct current stimulation targeting treatment refractory hallucinations and delusions: a traditional and accelerated stimulation case study
Source: Front Psychiatry. 2025 May 8;16:1568895. doi: 10.3389/fpsyt.2025.1568895 (PMC12095357; doi:10.3389/fpsyt.2025.1568895)

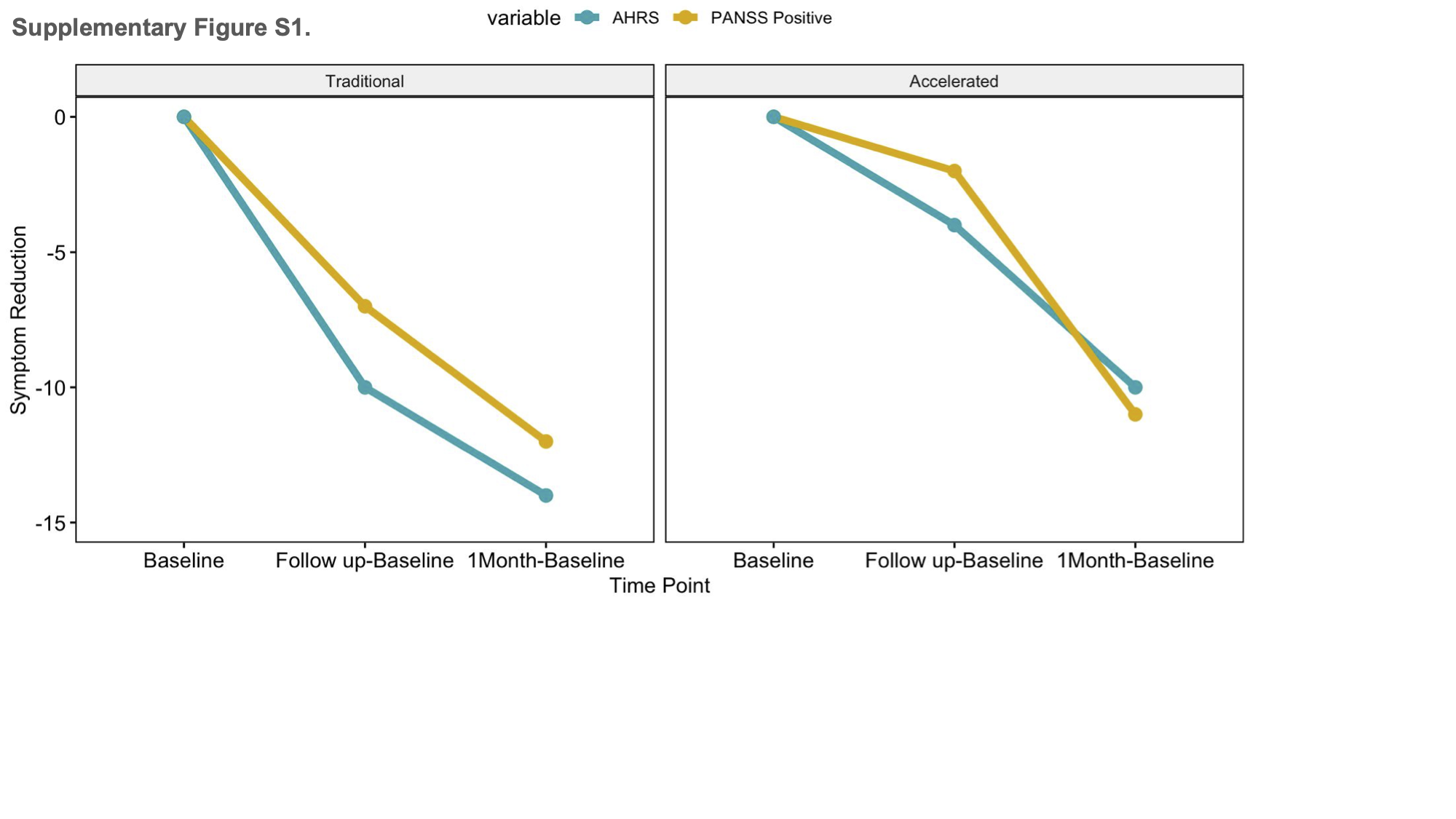

Supplement: Supplementary file 1 [file Image1.tiff]

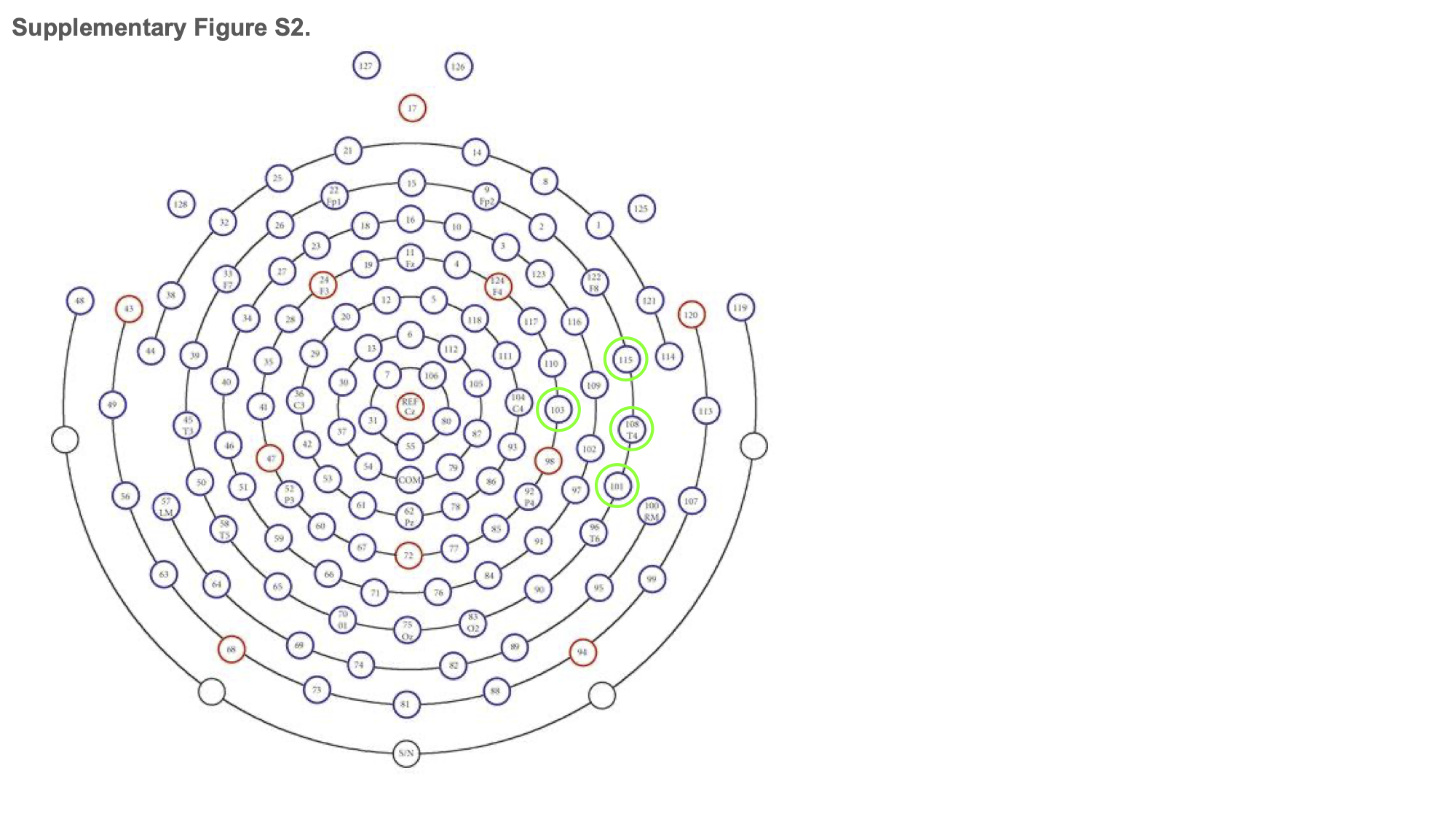

Supplement: Supplementary file 2 [file Image2.tiff]

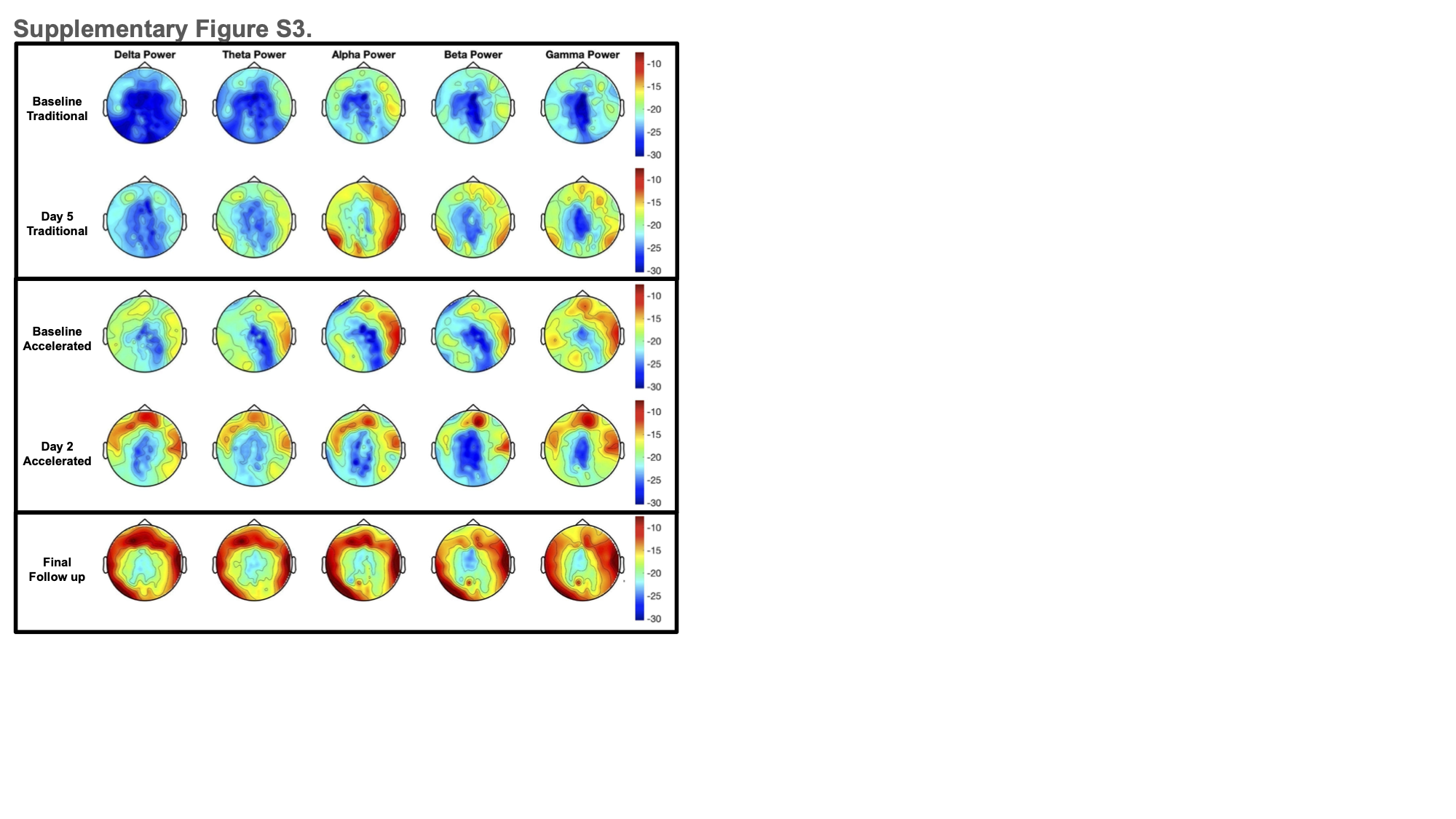

Supplement: Supplementary file 3 [file Image3.tiff]
