# Supplementary material for: Case Report: Lesion network guided transcranial direct current stimulation targeting treatment refractory hallucinations and delusions: a traditional and accelerated stimulation case study
Source: Front Psychiatry. 2025 May 8;16:1568895. doi: 10.3389/fpsyt.2025.1568895 (PMC12095357; doi:10.3389/fpsyt.2025.1568895)
Supplement: Supplementary file 4 [file Supplementaryfile1.docx]

**Supplementary Figure Legends:**

**Figure S1** Shows the changes scores calculated for AHRS and PANSS Positive variables express the magnitude of change over time for both Traditional and Accelerated protocols.

**Figure S2** Shows the selection of electrodes on the head to quantify absolute power for the ROI related to the rSTS.

**Figure S3** Portrays topographies of the absolute EEG power values extracted from the 5 minute, eyes open, resting state EEG for each time point in both the Traditional and Accelerated protocols as well as final follow up time point. NOTE: Auditory Hallucination Rating Scale (AHRS); Positive and Negative Symptom Scale (PANSS); Region of Interest (ROI); Right Superior Temporal Sulcus (rSTS).
